# Repeated psychological stress, chronic vicarious social defeat stress, evokes irritable bowel syndrome-like symptoms in mice

**DOI:** 10.3389/fnins.2022.993132

**Published:** 2022-10-06

**Authors:** Toshinori Yoshioka, Misaki Ohashi, Kenjiro Matsumoto, Tomoki Omata, Takumi Hamano, Mayuna Yamazaki, Sayaka Kimiki, Kotaro Okano, Riho Kobayashi, Daisuke Yamada, Noriyasu Hada, Shinichi Kato, Akiyoshi Saitoh

**Affiliations:** ^1^Laboratory of Pharmacology, Faculty of Pharmaceutical Sciences, Tokyo University of Science, Noda, Japan; ^2^Division of Pathological Sciences, Department of Pharmacology and Experimental Therapeutics, Kyoto Pharmaceutical University, Kyoto, Japan; ^3^Laboratory of Pharmacognosy, Faculty of Pharmaceutical Sciences, Tokyo University of Science, Noda, Japan

**Keywords:** gut-brain axis, social defeat stress, psychological stress, intestinal abnormality, irritable bowel syndrome, animal model

## Abstract

Increasing evidence has demonstrated that emotional states and intestinal conditions are inter-connected in so-called “brain–gut interactions.” Indeed, many psychiatric disorders are accompanied by gastrointestinal symptoms, such as the irritable bowel syndrome (IBS). However, the functional connection remains elusive, partly because there are few useful experimental animal models. Here, we focused on a highly validated animal model of stress-induced psychiatric disorders, such as depression, known as the chronic vicarious social defeat stress (cVSDS) model mice, which we prepared using exposure to repeated psychological stress, thereafter examining their intestinal conditions. In the charcoal meal test and the capsaicin-induced hyperalgesia test, cVSDS model mice showed a significantly higher intestinal transit ratio and increased visceral pain-related behaviors, respectively. These changes persisted over one month after the stress session. On the other hand, the pathological evaluations of the histological and inflammatory scores of naive and cVSDS model mice did not differ. Furthermore, keishikashakuyakuto—a kampo medicine clinically used for the treatment of IBS—normalized the intestinal motility change in cVSDS model mice. Our results indicate that cVSDS model mice present IBS-like symptoms such as chronic intestinal peristaltic changes and abdominal hyperalgesia without organic lesion. We therefore propose the cVSDS paradigm as a novel animal model of IBS with wide validity, elucidating the correlation between depressive states and intestinal abnormalities.

## Introduction

Irritable bowel syndrome (IBS) is a disorder chronically presenting gastrointestinal symptoms derived from the small and large intestines, even with no primary organic, systemic, or metabolic illness. According to ROME IV, IBS is categorized by stool consistency into four subtypes—IBS with constipation (IBS-C), IBS with diarrhea (IBS-D), mixed IBS (IBS-M), and unclassified IBS—and is generally characterized by the symptoms of chronic abnormal intestinal movement and abdominal pain without organic pathological changes (Lacy et al., 2016; Bai et al., 2017). However, the detailed pathophysiological conditions and underlying mechanism of IBS remain unclear, and effective treatment has not been established (Adriani et al., 2018).

Accumulating evidence has asserted a deep anatomical and functional brain and gut association—the “gut–brain axis” (Rhee et al., 2009; Mayer, 2011; Cryan and Dinan, 2012). Notably, many IBS patients have concurrent psychiatric disorders, such as depression, anxiety, and posttraumatic stress disorder (PTSD) (Palsson and Drossman, 2005; Ng et al., 2019). Correspondingly, for example, the representative symptoms of depression include not only depressed mood, anxiety, and anhedonia but also abdominalgia, constipation, and diarrhea (Ballou et al., 2019). Especially in digestive symptoms, in major depressive disorder (MDD) patients’ brain–gut interactions have adverse influences (Liang et al., 2018; Du et al., 2020). Emotional states and gut dysfunctions are thus considered to be highly associated.

One of the difficulties in elucidating the pathophysiology of IBS is the paucity of useful animal models. Recently, the chronic social defeat stress (cSDS) and chronic vicarious social defeat stress (cVSDS) models have been regarded as widely valid animal models of MDD and PTSD that also presents anxiety- and anhedonia-like phenotypes (Kudryavtseva et al., 1991; Rygula et al., 2005, 2006; Warren et al., 2013; Iñiguez et al., 2019). Notably, we previously reported the induction by the juvenile cSDS paradigm in mice of IBS-like symptoms in their adulthood (Matsumoto et al., 2021). While cSDS model mice (hereinafter referred to as “physical stress (PS) mice,” as in previous reports) are submitted to repeated physical attacks from the other mouse, cVSDS model mice [“emotional stress (ES) mice”] receive emotional stress only through witnessing PS mice (Sial et al., 2016). Recently, we found that chronic psychological stress of cVSDS significantly diminished the cell survival rate in the dentate gyrus of the hippocampus, which is closely related to the pathophysiological condition of depressive disorders (Yoshioka et al., 2022).

Here, we focused on the cVSDS paradigm and evaluated the impact of ESs on intestinal conditions. We further assessed the potential of the paradigm as a novel animal model of IBS.

## Materials and methods

### Animals

We obtained male C57BL/6J mice, aged 5–6 weeks, and “aggressor” CD-1 retired breeder mice from Sankyo Labo Service Corporation Inc. (Tokyo, Japan), and acclimatized them to the breeding room for about 7 days before the experiments. We housed the mice under controlled air temperature and pressure and 12-h light/dark cycles (lights on between 08:00 and 20:00) with *ad libitum* food and water. We housed C57BL/6J mice 4–6 mice per cage (225 × 338 × 140 mm), and CD-1 mice, singly. We conducted all experiments in accordance with the guidelines of the animal welfare committees at the Tokyo University of Science (Approval Nos. Y19032, Y20020, Y21002, and Y22014).

### Chronic social defeat stress and chronic vicarious social defeat stress paradigms

We performed the procedure for cSDS and cVSDS conditioning as previously reported with minor modifications (Sial et al., 2016; Yoshioka et al., 2022). Briefly, we divided mice randomly into three groups (naive; PS; ESs). We housed the “aggressor” CD-1 mice individually in their home cages and used them for experiments followed by a 3-day screening. During a defeat session, we placed an ES mouse on one side of the home cage of an “aggressive” CD-1 mouse separated by a perforated acryl divider, and then exposed a PS mouse to the CD-1 mouse. We performed this procedure for 10 min per day around 18:00–19:00 and repeated it over ten consecutive days. Each day we subjected each PS and each ES mouse to a different CD-1 mouse. After each session, we housed the PS mice with the CD-1 mice, separating each pair by the divider, and housed the ES mice singly until the next session; we housed the naive mice 4–6 in a cage for 10 days.

### Social interaction test

On day 11 counted from the first defeat session, we conducted a SIT to evaluate stress condition (Golden et al., 2011). We placed a mouse in the interaction field (450 mm × 450 mm) with a wire-mesh cage at one end. We tracked the movements of the mouse for 2.5 min before placing an unfamiliar “aggressor” CD-1 target mouse in the wire-mesh cage, and tracked the movements for another 2.5 min. We then auto-measured the time spent in the interaction zone (area around the wire-mesh cage, 140 mm × 240 mm) using SCANET-40 (Melquest Ltd., Toyama, Japan).

### Plasma corticosterone quantification

We collected a three-drop blood sample from the submandibular vein of mice with a 25-gauge needle. Immediately thereafter, we centrifuged the blood samples for 10 min at 2 000 × *g* at 4°C, and aliquoted the supernatant for the enzyme-linked immunosorbent assay, storing it at -20°C until use. We measured corticosterone levels with a Corticosterone ELISA Kit (Enzo Life Sciences Inc., Farmingdale, NY, USA) according to the manufacturer’s protocol.

### Charcoal meal test

We deprived mice of food for 14–16 h before the test. Following another single cSDS or cVSDS exposure for 10 min, we orally administrated 10 mL/kg of vermilion Indian ink, sacrificed the mice 10 min thereafter by dislocating the cervical vertebra, and immediately extracted the duodenum and small intestine (from the end of the pylorus to the origin of cecum). We calculated the intestinal transit ratio as the charcoal transport distance divided by the total intestine length.

### Defecation frequency and stool water content

We conducted stool evaluation according to a previous report (Li et al., 2006). In brief, we placed each mouse in a clean cage for 1 h and collected fecal pellets in a sealed sample tube each time when the animals defecated. After counting the number of pellets and weighing the wet stool, we dried the stool overnight at 65°C and weighed the dry stool as well. The stool water content was calculated as the difference between the wet and dry stool weights divided by the wet stool weight.

### Capsaicin-induced hyperalgesia test

We performed this test with minor modifications to that of a previous report (Eijkelkamp et al., 2007). In brief, we, respectively, habituated mice in an acryl cylinder (diameter, 115 mm; height, 180 mm) for 1 h. We then administered 0.1 mL of capsaicin-containing reagent (0.1% wt/vol in 4% Tween80/0.9% NaCl; FUJIFILM Wako Pure Chemical, Osaka, Japan) intrarectally, and counted the number of visceral pain-related behaviors for 15 min. We defined the behavioral evaluation criteria as follows: licking (of the lower abdomen), squashing (of the abdomen against the floor), and jumping (vertically). We also counted immobility when the mice stopped all behaviors apart from respiration movement for more than 3 sec per 5 sec duration.

### Pathological evaluation

We extracted the small and large intestines from the mice, washed them with phosphate buffered saline, fixed them in a neutral buffered 10% formalin solution at 4°C, embedded them into paraffin, sectioned them at a thickness of 4 μm, and stained them with hematoxylin and eosin using a standard protocol. We carried out the histological scoring based on previous reports (Theiss et al., 2009; Matsumoto et al., 2018).

### Intestinal permeability

We evaluated intestinal permeability as described previously (Matsumoto et al., 2021). In brief, we collected blood from the submandibular vein of mice 1 h after the oral administration of 200 μL of FITC-dextran (MW 4000) solution (50 mg/mL; Merck KGaA, Darmstadt, Germany). Immediately thereafter, we centrifuged the blood samples for 10 min at 2000 × *g* and 4°C and aliquoted the supernatant. We measured the plasma FITC concentration as fluorescence intensity using ALVO MX (PerkinElmer, Waltham, MA, USA).

### Drug treatment

We prepared keishikashakuyakuto with hot water extraction and obtained it as a freeze-dried mixture of Cinnamon Bark (lot. 6G28M) 4: Peony Root (lot. 8E24M) 6: Jujube (lot. 8E01M) 4: Glycyrrhiza (lot. 8C27) 2: Ginger (lot. 8E15M) 1. We purchased all cut crude ingredients from Daikoshoyaku Co., Ltd. (Aichi, Japan). We dissolved the drug in 0.1% methylcellulose, and 30 min before the test, we orally administered mice the drug at a dose of 1 g/kg.

### Statistical analysis

We determined the sample size by referring to previous reports (Eijkelkamp et al., 2007; Warren et al., 2013; Matsumoto et al., 2021; Yoshioka et al., 2022). Data were acquired from at least two divided experiments. Investigators were blinded to animal groups and/or drug administration information during testing. All data are presented as means ± standard error of the mean (s.e.m.). We performed the analysis using GraphPad Prism7 (GraphPad Software Inc., San Diego, CA, USA). We analyzed the data from two groups using the Student’s *t*-test. We analyzed the data from the remaining groups using one-way or two-way analysis of variance (ANOVA), followed by the *post hoc* Bonferroni’s test. We defined statistical significance as *p < 0.05, ^**^p < 0.01.

## Results

### Preparation of physical stress and emotional stress mice

**FIGURE 1:**
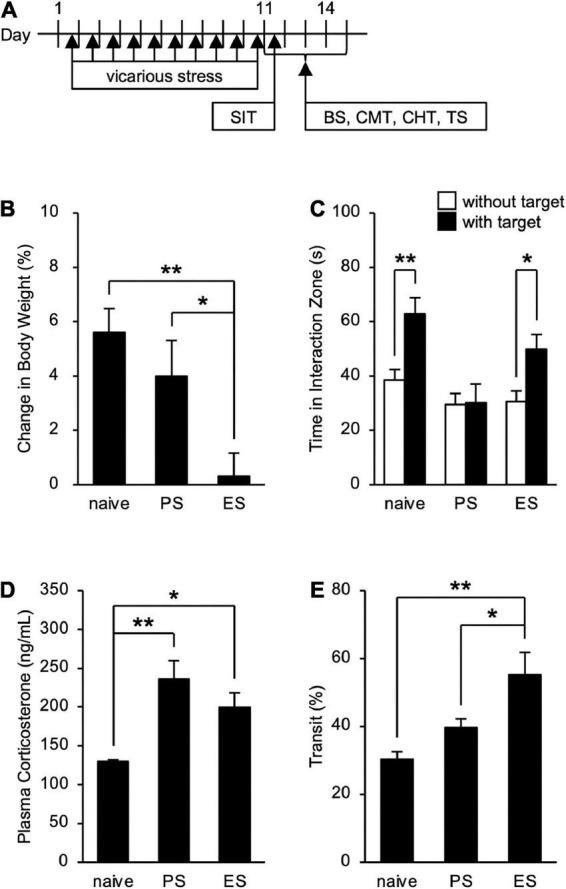
Effects of chronic social defeat stress (cSDS) and chronic vicarious social defeat stress (cVSDS) on the physiological and behavioral responses of mice. **(A)** Schematic representations of experimental designs. SIT: social interaction test; BS: blood sampling; CMT: charcoal meal test; CHT: capsaicin-induced hyperalgesia test; TS: tissue sampling. **(B)** Effect of cSDS and cVSDS on body weight gain. Data are presented as means ± s.e.m., and analyzed using one-way factorial ANOVA, followed by Bonferroni’s *post hoc* test. *p < 0.05, **p < 0.01. naive, *n* = 24; PS, *n* = 24; ES, *n* = 28. **(C)** Time spent in the interaction zone in the social interaction test. Data are presented as means ± s.e.m., and analyzed using two-way factorial ANOVA, followed by Bonferroni’s *post hoc* test. *p < 0.05, **p < 0.01. naive, *n* = 20; PS, *n* = 22; ES, *n* = 23. **(D)** Plasma corticosterone quantification. Data are presented as means ± s.e.m., and analyzed using one-way factorial ANOVA, followed by Bonferroni’s *post hoc* test. *p < 0.05, **p < 0.01. naive, *n* = 6; PS, *n* = 6; ES, *n* = 7. **(E)** Intestinal transit ratio in the charcoal meal test. Data are presented as means ± s.e.m., and analyzed using one-way factorial ANOVA, followed by Bonferroni’s *post hoc* test. *p < 0.05, **p < 0.01. naive, *n* = 12; PS, *n* = 15; ES, *n* = 15.

To assess the influence of cSDS and cVSDS on the physiological and behavioral responses, we designed an experimental plan indicated in Figure 1A. In evaluating stress condition, our results indicated that cVSDS, but not cSDS, significantly impaired body weight gain of mice compared to naive mice (one-way ANOVA; *F*_(2_, _73)_ = 7.332, *p* < 0.01; Figure 1B). In the SIT, cSDS, but not cVSDS, significantly reduced the time spent in the interaction zone in the presence of an unfamiliar CD-1 target mouse (two-way ANOVA; between-group main effect: *F*_(2_, _124)_ = 7.720, *p* < 0.01; within-group main effect: *F*_(1_, _124)_ = 11.94, *p* < 0.01; interaction effects: *F*_(2_, _124)_ = 2.888, non-significant; Figure 1C). On the other hand, plasma corticosterone level was increased in both paradigms (one-way ANOVA; *F*_(2_, _16)_ = 8.969, *p* < 0.01; Figure 1D). These results closely resemble those of previous reports (Warren et al., 2013; Yoshioka et al., 2022).

### Chronic vicarious social defeat stress, but not chronic social defeat stress, increases intestinal peristalsis

**FIGURE 2:**
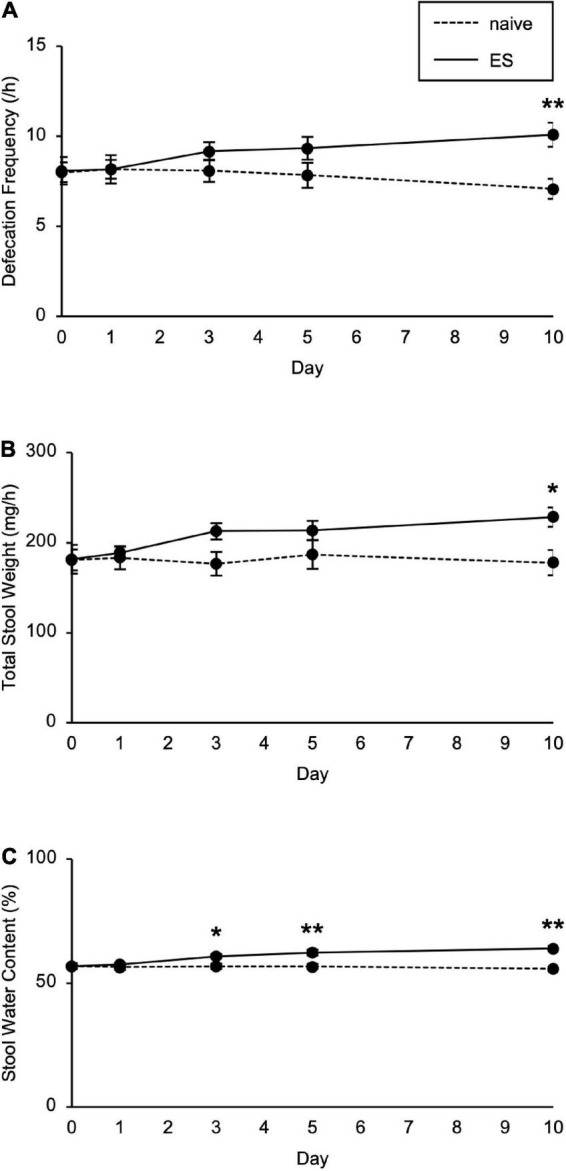
Chronic vicarious social defeat stress increased defecation frequency and stool water content. **(A–C)** Defecation frequency **(A)**, total stool weight **(B)**, and stool water content **(C)** for 1 h on different days during the stress session. Data are presented as means ± s.e.m. Data were analyzed using two-way repeated measures ANOVA, followed by Bonferroni’s *post hoc* test. *p < 0.05, **p < 0.01. *n* = 12 for each group.

We next investigated the impact of cSDS and cVSDS on intestinal peristalsis. After the 10-day stress loading, the charcoal transit ratio in ES mice, but not PS mice, was significantly elevated compared to that of naive mice in the CMT (one-way ANOVA; *F*_(2_, _39)_ = 7.719, *p* < 0.01; Figure 1E), indicating that cVSDS, but not cSDS, influenced intestinal peristalsis. However, in the SIT, while a single VSDS exposure did not affect the time spent in the interaction zone in the absence or presence of an unfamiliar CD-1 target mouse (two-way ANOVA; between-group main effect: *F*_(1_, _28)_ = 0.001874, non-significant; within-group main effect: *F*_(1_, _28)_ = 1.926, non-significant; interaction effects: *F*_(1_, _28)_ = 0.2629, non-significant; Supplementary Figure 1A), the charcoal transit ratio was decreased (Student’s *t*-test: *p* = 0.0454; Supplementary Figure 1B). In addition, ES mice showed a gradual increase in the defecation frequency, total stool weight, and stool water content during the stress session (two-way ANOVA; main effect of stress: *F*_(1_, _22)_ = 4.842, *p* = 0.0386, *F*_(1_, _22)_ = 6.520, *p* = 0.0181, *F*_(1_, _22)_ = 11.77, *p* < 0.01, respectively; main effect of time: *F*_(4_, _88)_ = 0.4411, non-significant, *F*_(4_, _88)_ = 1.264, non-significant, *F*_(4_, _88)_ = 11.16, *p* < 0.01, respectively; interaction effects: *F*_(4_, _88)_ = 2.203, non-significant, *F*_(4_, _88)_ = 1.614, non-significant, *F*_(4_, _88)_ = 15.94, *p* < 0.01, respectively; Figure 2), suggesting that cVSDS evokes diarrhea-like symptoms in mice.

### Chronic vicarious social defeat stress evokes abdominal hyperalgesia

**FIGURE 3:**
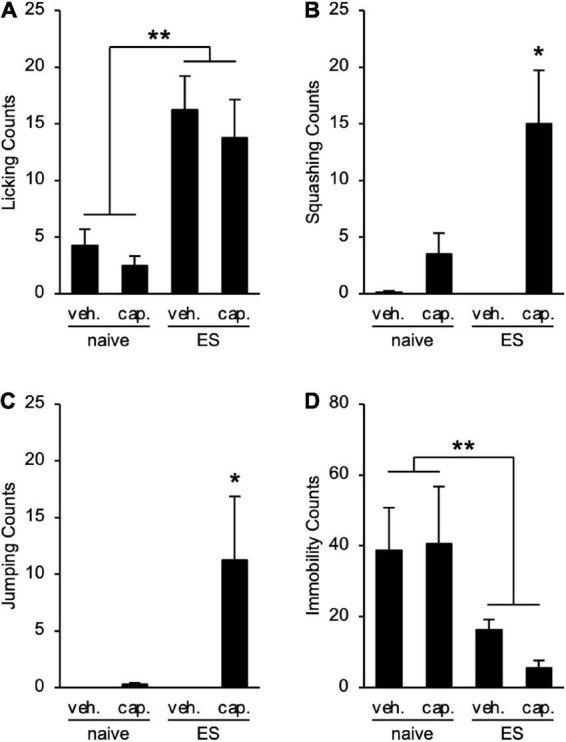
Chronic vicarious social defeat stress induces abdominal hyperalgesia. **(A–D)** Visceral pain-related behavior counts in the capsaicin-induced hyperalgesia test. **(A)** Licking; **(B)** Squashing; **(C)** Jumping; **(D)** Immobility. veh.: vehicle; cap.: capsaicin. Data are presented as means ± s.e.m., and analyzed using two-way factorial ANOVA, followed by Bonferroni’s *post hoc* test. *p < 0.05, **p < 0.01. *n* = 8 for each group.

To examine the visceral pain in ES mice, we performed the CHT. cVSDS provoked an increase in licking counts (two-way ANOVA; main effect of stress: *F*_(1_, _28)_ = 23.57, *p* < 0.01; main effect of drug: *F*_(1_, _28)_ = 0.7875, non-significant; interaction effects: *F*_(1_, _28)_ = 0.02452, non-significant; Figure 3A), and capsaicin administration increased squashing and jumping counts in ES mice (two-way ANOVA; main effect of stress: *F*_(1_, _28)_ = 5.036, *p* = 0.0329, *F*_(1_, _28)_ = 3.848, non-significant, respectively; main effect of drug: *F*_(1_, _28)_ = 13.14, *p* < 0.01, *F*_(1_, _28)_ = 4.206, *p* = 0.0498, respectively; interaction effects: *F*_(1_, _28)_ = 5.260, *p* = 0.0295, *F*_(1_, _28)_ = 3.848, non-significant, respectively; Figures 3B,C), suggesting that cVSDS induces abdominal hyperalgesia. On the other hand, we noted a decrease in immobility counts in ES mice regardless of administration of capsaicin (two-way ANOVA; main effect of stress: *F*_(1_, _28)_ = 11.88, *p* < 0.01; main effect of drug: *F*_(1_, _28)_ = 0.0003423, non-significant; interaction effects: *F*_(1_, _28)_ = 0.02772, non-significant; Figure 3D).

### Chronic vicarious social defeat stress did not change intestinal pathological conditions

**FIGURE 4:**
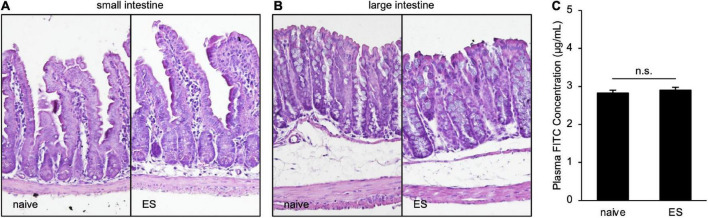
Chronic vicarious social defeat stress does not affect pathological states. **(A,B)** Representative images of hematoxylin–eosin staining of small intestine **(A)** and large intestine **(B)**. Histological and inflammatory statuses are evaluated by epithelial surface damage, crypt damage, the number of inflammatory cells, and presence of ulceration. **(C)** Plasm FITC concentration 1 h after the administration of FITC-dextran (MW 4000) on Day 11. Data are presented as means ± s.e.m., and analyzed using Student’s *t*-test. *n* = 12 for each group.

To clarify the histological status in ES mice, we performed hematoxylin–eosin staining of the small and large intestines. Scores of the epithelial surface damage, crypt damage, the number of inflammatory cells, and the presence of ulceration did not differ between naive and ES mice (Figures 4A,B; Supplementary Table 1), indicating that cVSDS did not alter pathological states. Moreover, the plasma concentration of FITC-dextran, which was administrated orally, was same in ES mice and naive mice (Student’s *t*-test: non-significant; Figure 4C), suggesting that cVSDS did not influence intestinal permeability in mice.

### Long-term effects of chronic vicarious social defeat stress on intestinal hypermotility and abdominal analgesia

**FIGURE 5:**
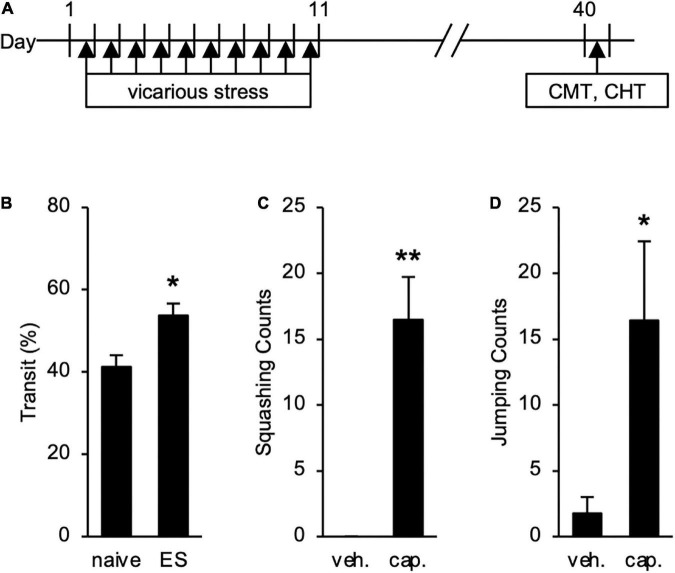
Long-term Effects of chronic vicarious social defeat stress (cVSDS) on intestinal peristalsis and abdominal hyperalgesia. **(A)** Experimental design to evaluate long-term effects of cVSDS. CMT: charcoal meal test; CHT: capsaicin-induced hyperalgesia test. **(B)** Intestinal transit ratio in the charcoal meal test. Data are presented as means ± s.e.m., and analyzed using Student’s *t*-test. *p < 0.05. naive, *n* = 6; ES, *n* = 5. **(C,D)** Visceral pain-related behavior counts in the capsaicin-induced hyperalgesia test. **(C)** Squashing; **(D)** Jumping. veh.: vehicle; cap.: capsaicin. Data are presented as means ± s.e.m., and analyzed using two-way factorial ANOVA, followed by Bonferroni’s *post hoc* test. *p < 0.05, **p < 0.01. naive, *n* = 12; ES, *n* = 12.

We also tested long-term effects of cVSDS on intestinal conditions (Figure 5A). The increased transit ratio in the CMT persisted (Student’s *t*-test: *p* = 0.0145; Figure 5B), and squashing and jumping behaviors in the CHT were still observed (Student’s *t*-test: *p* < 0.01, *p* = 0.0258, respectively; Figures 5C,D) 30 days after the stress loading in ES mice. These results suggest that ES mice present chronic abdominal abnormalities.

### Keishikashakuyakuto normalized the chronic vicarious social defeat stress-induced intestinal hypermotility

**FIGURE 6:**
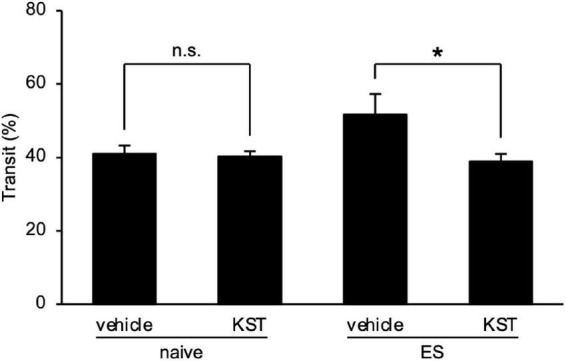
Keishikashakuyakuto normalizes chronic vicarious social defeat stress-induced intestinal peristaltic change. Keishikashakuyakuto (KST; 1 g/kg, p.o.) attenuated the intestinal transit ratio in ES mice, but not in naive mice, in the charcoal meal test on day 12. Data are presented as means ± s.e.m., and analyzed using two-way factorial ANOVA, followed by Bonferroni’s *post hoc* test. *p < 0.05. naive, *n* = 10; ES, *n* = 6.

Finally, we determined the effect of keishikashakuyakuto, which is used clinically to treat IBS, on the intestinal motility changes in ES mice. Two days after cVSDS, keishikashakuyakuto decreased the charcoal transit ratio in ES mice in the CMT, but did not affect that of naive mice (two-way ANOVA; main effect of stress: *F*_(1_, _28)_ = 2.797, non-significant; main effect of drug: *F*_(1_, _28)_ = 5.702, *p* = 0.0239; interaction effects: *F*_(1_, _28)_ = 4.508, *p* = 0.0427; Figure 6). These results suggest that keishikashakuyakuto modulates intestinal motility.

## Discussion

In the current research, we investigated the intestinal conditions of mice exposed to repeated ESs in the cVSDS paradigm, which has played a crucial role in recent preclinical studies as an animal model of MDD and PTSD because of its wide validity in three modes: constructive, face, and predictive (Sial et al., 2016; Yoshioka et al., 2022). We first confirmed that cSDS and/or cVSDS affect physiological and behavioral parameters—such as body weight gain, social behaviors, and plasma corticosterone concentration. Our findings were consistent with previous reports (Warren et al., 2013; Yoshioka et al., 2022). We also found that cVSDS rather than cSDS increased the intestinal transit ratio in the CMT, an effect that persisted 1 month after stress loading, defecation frequency, and stool water content. ES mice also showed visceral pain-related behaviors in the CHT both immediately and one month after exposure to stress. On the other hand, the pathological status and intestinal permeability of the intestine of naive and ES mice did not differ. These results suggest that cVSDS in mice provokes IBS-D-like symptoms such as chronic intestinal peristaltic exacerbations and abdominal hyperalgesia without intestinal lesions. Furthermore, treatment with an IBS-therapeutic, keishikashakuyakuto, induced recovery of the observed intestinal transport abnormality. We therefore propose the cVSDS paradigm as a new animal model of IBS with wide validity.

Conventional animal models of IBS are limited by their requirement for preparation with an acute PS, such as restraint stress, or drug-induced intestinal inflammation (Williams et al., 1988; Qin et al., 2011; Larauche et al., 2012). Besides, other animal models, such as neonatal maternal separation and chronic water avoidance stress paradigms, present intestinal pathological changes (Söderholm et al., 2002; Bradesi et al., 2005; Vannucchi and Evangelista, 2018). Here, we revealed that repeated psychological stress-induced IBS-D-like symptoms without morphological changes and inflammations of the intestine, conferring on this cVSDS paradigm higher constructive and face validities. From the aspect of the gut–brain axis, we consider the hypothesis that the insular cortex plays an important role in determining the phenotype of ES mice as the insular cortex is a part of the upper central nervous system controlling digestive functions and is involved in the process of coping with psychological stress. Indeed, it was indicated in some rodent studies that psychological stress activates the insular cortex (Nakatake et al., 2020; Zhang et al., 2022). Therefore, elucidating the functional mechanism underlying IBS-like symptoms in ES mice would substantially lead to understanding the “gut–brain axis.” Moreover, how different kinds of stress and individual differences affect intestinal functions is not well known both preclinically and clinically; therefore, creating an IBS-C animal model is keenly anticipated.

We previously reported that cSDS in juvenile mice causes IBS-like symptoms in their adulthood, an animal model of IBS now regarded as both novel and highly validated (Matsumoto et al., 2021). We consider both the cSDS and the cVSDS paradigms to be very useful animal models of IBS in the elucidation of the pathophysiological conditions and the design of therapeutic drugs. Remarkably, early childhood cSDS evokes symptoms at a later stage, while cVSDS does so at either the initial or the later stage; further studies are required to clarify the deferment of onset time. Interestingly, our present result indicates that cSDS did not significantly affect intestinal peristalsis immediately after the stress loading, and a single VSDS contrarily suppressed the motility. This may be due to the dominance of the sympathetic nervous system in certain kinds of stress. In the cSDS paradigm, for instance, PS mice showed higher daily consumption of water compared to naive mice (data not shown), suggesting the possibility that cSDS evoked dipsia by activating the sympathetic nervous system. In other words, the possible dominance of the sympathetic nervous system in PS mice may have suppressed the increase in intestinal transit rate. Further studies are needed to determine the influences of cSDS and cVSDS on the autonomic nervous system.

As aforementioned, IBS patients show recurrent abdominal pain. Here, besides licking of the lower abdomen behaviors induced by cVSDS, ES mice presented squashing and jumping movements in the CHT both immediately and one month after cVSDS, suggesting repeated psychological stress-induced chronic abdominal hyperalgesia. Although previous reports have regarded immobility in the CHT as a visceral pain-related behavior (Eijkelkamp et al., 2007), in ES mice it was significantly low despite administration of capsaicin, because ES mice showed hyperactivity (Yoshioka et al., 2022). We thus propose that ES mice exhibited IBS-D-like symptoms with persistent abdominal pain.

In Oriental medicine, keishikashakuyakuto is an effective treatment of functional gastrointestinal disorders, commonly used for all subtypes of IBS (Saitoh et al., 1999; Oka et al., 2014; Higurashi et al., 2018; Kimura, 2019). According to our results, keishikashakuyakuto controlled the increased intestinal peristalsis in ES mice but did not affect naive mice, suggesting that keishikashakuyakuto not only suppressed intestinal motility but also improved stress-induced diarrhea-like symptom. Hence, we confirmed the predictive validity of the cVSDS paradigm as an animal model of IBS. Additionally, although keishikashakuyakuto is prescribed to relieve abdominal pain (Oka et al., 2014), visceral pain-related behaviors in ES mice tested using the CHT deteriorated with the administration of keishikashakuyakuto (data not shown). This result suggests the possibility that keishikashakuyakuto increases 1) retentivity and/or 2) reactivity of capsaicin in the colon, because keishikashakuyakuto has smooth muscle relaxant actions and raises the extracellular concentration of calcium ions, a key mediator of the capsaicin receptor, transient receptor potential vanilloid 1 (Takayama et al., 2015; Lee et al., 2020). Thus, designing another test system would be required to evaluate the effects of keishikashakuyakuto on abdominalgia.

To date, many IBS animal models have induced organic inflammation in the intestine or have been accompanied by physical stress (Williams et al., 1988; Söderholm et al., 2002; Bradesi et al., 2005; Qin et al., 2011; Larauche et al., 2012; Vannucchi and Evangelista, 2018). Patients with IBS symptoms—for which psychological stress is considered to be a major cause—are often refractory to treatment, amplifying the need to elucidate its pathophysiology. We propose that the present model—induced only by psychological stress—will be an unprecedented and useful animal model.

In conclusion, we demonstrate here for the first time that cVSDS-induced psychological stress alone causes IBS-D-like symptoms in mice. Therefore, we propose the potential of the cVSDS paradigm as a novel unique animal model of IBS with constructive, face, and predictive validities.

## Data availability statement

The original contributions presented in this study are included in the article/Supplementary material, further inquiries can be directed to the corresponding author/s.

## Ethics statement

The animal study was reviewed and approved by the animal welfare committees at the Tokyo University of Science.

## Author contributions

TY and MO performed most of the work. KM, TO, TH, MY, SKi, KO, and RK performed the experiments. KM, DY, NH, and SKa provided technical and intellectual advice. AS supervised the project. All authors contributed to the article, agreed to be accountable for all aspects of the work, and approved the submitted version.
