# Supplementary material for: Repeated psychological stress, chronic vicarious social defeat stress, evokes irritable bowel syndrome-like symptoms in mice
Source: Front Neurosci. 2022 Oct 6;16:993132. doi: 10.3389/fnins.2022.993132 (PMC9582264; doi:10.3389/fnins.2022.993132)
Supplement: Supplementary file 1 [file Data_Sheet_1.PDF]

Supplementary Material

1 Supplementary Figure 1

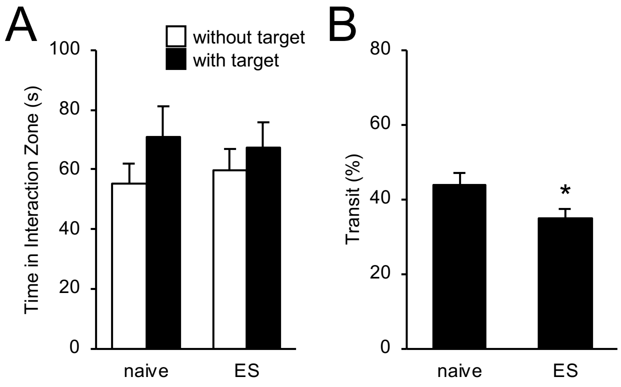

Supplementary Figure 1. Acute vicarious social defeat stress suppresses intestinal peristalsis. (**A**) Time spent in the interaction zone in the social interaction test. Data are presented as means ± s.e.m., and analyzed using two-way factorial ANOVA. naive, n = 8; ES, n = 8. (**B**) Intestinal transit ratio in the charcoal meal test. Data are presented as means ± s.e.m., analyzed using Student's t-test. *p < 0.05. naive, n = 8; ES, n = 8.

2 Supplementary Table 1

small intestine

naive	epithelium	crypt	inflammation	ulceration	ES	epithelium	crypt	inflammation	ulceration
1	0	0	0	0	1	0	0	0	0
2	0	0	0	0	2	0	0	0	0
3	0	0	0	0	3	0	0	0	0
4	0	0	0	0	4	0	0	0	0
5	0	0	0	0	5	0	0	1	0
6	0	0	0	0	6	0	0	0	0

large intestine

naive	epithelium	crypt	inflammation	ulceration	ES	epithelium	crypt	inflammation	ulceration
1	0	0	0	0	1	0	0	0	0
2	0	0	0	0	2	0	0	0	0
3	0	0	0	0	3	0	0	0	0
4	0	0	0	0	4	0	0	1	0
5	0	0	0	0	5	0	0	0	0
6	0	0	0	0	6	0	0	0	0

Supplementary Table 1. Scores in the pathological evaluation of the small intestine and large intestine in chronic vicarious social defeat stress paradigm.
